# Supplementary material for: Origin of New Lineages by Recombination and Mutation in Avian Infectious Bronchitis Virus from South America
Source: Viruses. 2022 Sep 21;14(10):2095. doi: 10.3390/v14102095 (PMC9609748; doi:10.3390/v14102095)
Supplement: Supplementary file 1 [file viruses-14-02095-s001.zip › viruses-1862224-supplementary.pdf]

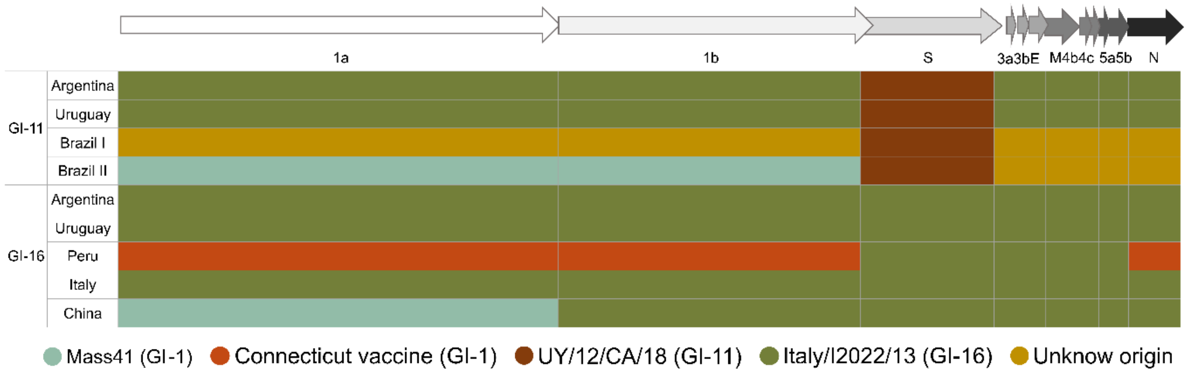

Figure S1. Genomic organization of the GI-16 and GI-11 strains. The ORFs analyzed are indicated with different shades of grey. The identity of each ORF is indicated for the different GI-11 and GI-16 strains according to their similarity with IBV reference genomes (shown with different colors).
